# Integrated transcriptomic and metabolomic analyses of the molecular mechanisms of two highland barley genotypes with pyroxsulam responses

**DOI:** 10.3389/fpls.2022.1030578

**Published:** 2022-12-21

**Authors:** Hua Weng, Jiahui Yan, Liangzhi Guo, Hongyu Chen

**Affiliations:** State Key Laboratory of Plateau Ecology and Agriculture, Scientific Observing and Experimental Station of Crop Pest in Xining, Ministry of Agriculture, Key Laboratory of Agricultural Integrated Pest Management of Qinghai Province, Qinghai Academy of Agriculture and Forestry Sciences, Xining, China

**Keywords:** flavonoids, antioxidants, transcriptome, metabolome, highland barley, pyroxsulam

## Abstract

Highland barley is one of the few crops that can be grown at high elevations, making it a key resource within the Tibet Plateau. Weeds are a significant threat to highland barley production, and new herbicides and tolerant barley varieties are needed to control this ever-growing problem. A better understanding of existing herbicide resistance mechanisms is therefore needed. In this study, transcriptomic and metabolomic analyses were used to identify molecular and physiological changes in two highland barley genotypes with differing sensitivities to the herbicide pyroxsulam. We identified several stress-responsive metabolites, including flavonoids and antioxidants, which accumulated to significantly higher levels in the pyroxsulam-resistant genotype. Additionally, we found key genes in both the flavonoid biosynthesis pathway and the antioxidant system that were up-regulated in pyroxsulam-resistant barley. This work significantly expands on the current understanding of the molecular mechanisms underlying differing pyroxsulam tolerance among barley genotypes and provides several new avenues to explore for breeding or engineering tolerant barley.

## Introduction

Highland barley (*Hordeum vulgare* L.) is an economically important crop and is the only cereal which can mature normally in the short growing seasons often found at high altitudes (He and Jia, 2008). It is the fourth most important cereal crop worldwide and is primarily utilized for animal feed, brewing, distilling, and malting (Baik and Ullrich, 2008). Highland barley remains a major food source in some areas, such as West Asia and North Africa (Newman and Newman, 2006). Although it is grown throughout the world, the majority of its cultivation lies in East Asian countries, such as China, which produces 77% of the total highland barley grown worldwide (Li et al., 2011). Highland barley is also consumed as a staple crop in regions of the Qinghai-Tibet Plateau, including Sichuan, Gansu, Qinghai, and Tibet. Highland barley is consumed in this region due to its health benefits, which stem from its metabolites, bioactive carbohydrates, polyphenols, vitamins, phenolic, flavonoids, and β-glucans (Obadi et al., 2021). Additionally, research has indicated that highland barley may possess antihyperglycemic, antihyperlipidemic, and anticancer activities (Idehen et al., 2017). Despite these benefits, there are several challenges in growing barley in the Tibet Plateau, including invasive weeds that cause significant annual yield losses. The lack of herbicides which target these weeds without negatively impacting barley severely limits the development of the barley industry. The development of herbicide-resistant barley varieties is critical to increasing the production efficiency of this crop system by enabling the use of more efficient weed control systems.

Pyroxsulam is a new herbicide which is being widely used to prevent the growth of broad-leaved weeds in wheat fields because of its low quantity of application and high efficiency, was widely promoted in fields since being introduced to the herbicide market. Pyroxsulam is a new triazolopyrimidine sulfonamide acetolactate synthase (ALS)-inhibiting herbicide developed by Dow AgroSciences (Indianapolis, IN), which provides broad-spectrum control of many annual, biannual, and perennial weeds. It has the advantages of high efficacy with low doses and a favorable environmental profile (Jabusch and Tjeerdema, 2008). Pyroxsulam acts by inhibiting ALS, an important target for herbicides, which is an essential enzyme that catalyzes the first step in the synthesis of the branched-chain amino acids valine, leucine, and isoleucine (Gonzalez et al., 2008). The resulting lack of amino acids in the plant inhibits DNA synthesis, subsequently stopping cell division and causing death in susceptible plants (Fischer, 2019). Due to intensive use of ALS herbicides, some weeds have evolved resistance. Most reported cases of pyroxsulam-tolerant weeds involve mutations in the ALS gene, which result in changes at one of eight amino acid positions (Ala-122, Pro-197, Ala-205, Asp-376, Arg-377, Trp-574, Ser-653, and Gly-654) (Powles and Yu, 2010; Lan et al., 2022). Commercial formulations of pyroxsulam with the herbicide safener cloquintocet allow for selective weed control in cereal crops. Pyroxsulam is currently one of the most widely used herbicides in the world, but little is known about its effects on highland barley. A better understanding of the mechanisms underlying variable pyroxsulam tolerance among highland barley genotypes is critical to efficiently utilizing this herbicide in its production.

The success of breeding herbicide-tolerant varieties relies on the availability of tolerant locally adapted germplasm. Recently, the integration of various omics technologies has proven to be indispensable for determining potential tolerance mechanisms. We performed a comparative analysis of two highland barleys with differing tolerance to pyroxsulam and identified substantial differences in their metabolomes and transcriptomes. These differences included key genes in pathways associated with amino acid biosynthesis, reactive oxygen tolerance, and flavonoids, indicating that these pathways are responsible for the varying pyroxsulam tolerance among different barely varieties. Our results imply that pyroxsulam-tolerant highland barley varieties induce the expression of flavonoids and reactive oxygen species (ROS) scavenging enzymes in order to resist pyroxsulam toxicity. These results provide a good starting point for understanding highland barley pyroxsulam tolerance, although the underlying mechanisms are complex and require additional research. A deeper insight into the molecular mechanisms underlying barley pyroxsulam tolerance may enable the breeding of more tolerant barley varieties.

## Materials and methods

### Plant materials

Qing 0160 and Qing 0305 barley varieties were collected from Xining City, Qinghai Province, China (36.434419 N, 101.450759 E). The samples were identified by Liling Jiang and deposited at the National duplicate Genbank for crops, Xining, Qinghai Province, China. The specimen accession numbers were ZDM1651 and ZDM8091.

### Sample preparation and RNA isolation

Two highland barley genotypes were used for this study, including one pyroxsulam-sensitive genotype and one pyroxsulam-insensitive genotype. All highland barley seedlings were grown in a potting medium under a 11 h light (25°C)/13 h dark (20°C) day/night cycle with 75% humidity. Pyroxsulam herbicide was applied during the two-leaf stage, with the typical field application concentration of 12.5 g/666.7 m^2^. Leaf samples were harvested at zero, one, and six days after treatment with pyroxsulam, and then stored at −80°C for metabolite profiling and RNA-sequencing. Total RNA was isolated using an RNA extraction kit from Sangon (Shanghai, China).

### Library preparation for transcriptome sequencing

Total RNA was extracted from barley. The mRNA was then purified from total RNA using poly-T oligo-attached magnetic beads. Fragmentation was carried out using divalent cations under elevated temperature in First Strand Synthesis Reaction Buffer (5X). First-strand cDNA was synthesized using a random hexamer primer and M-MuLV Reverse Transcriptase, followed by RNaseH RNA digestion. Second-strand cDNA synthesis was subsequently performed using DNA Polymerase I and dNTPs. The remaining overhangs were converted into blunt ends *via* exonuclease/polymerase activities. After adenylation of the 3’ end of the DNA fragments, adaptors with hairpin loop structures were ligated to prepare for hybridization. In order to select cDNA fragments that were 370-420 bp in length, the library fragments were purified with the AMPure XP system (Beckman Coulter, Beverly, USA). Then, PCR was performed with Phusion High-Fidelity DNA polymerase, universal PCR primers, and an index (X) primer. Finally, PCR products were purified using the AMPure XP system, and library quality was assessed on an Agilent Bioanalyzer 2100.

### Sequence data mapping and transcriptome analysis

Raw FASTQ-formatted reads were first processed through in-house Perl scripts to generate clean reads, with adaptor sequences trimmed and reads containing Ns or low-quality bases removed. Scripts were then used to calculate Q20, Q30, and GC content. All downstream analyses were based on clean data, with only high-quality reads retained.

Reference genome and gene model annotation files were downloaded from a public database National Center for Biotechnology Information. The reference genome index was built using Hisat2 v2.0.5, and paired-end clean reads were aligned to the reference genome using Hisat2 v2.0.5.

### Differential expression analysis

Differential expression analysis of the two conditions (three biological replicates per condition) was performed using the DESeq2 R package v1.20.0. The resulting *P*-values were adjusted using Benjamini and Hochberg’s approach for controlling the false discovery rate. Genes with an adjusted *P*-value <0.05 identified by DESeq2 were considered differentially expressed.

### GO and KEGG enrichment analyses of differentially expressed genes

Gene Ontology (GO) enrichment analysis of differentially expressed genes (DEGs) was *#implemented *via* the clusterProfiler R package. GO terms with corrected *P*-values less than 0.05 were considered significantly enriched. KEGG was employed to determine high-level functions of DEGs (http://www.genome.jp/kegg/) (Kanehisa and Goto, 2000). We used the clusterProfiler R package to test the statistical enrichment of DEGs in KEGG pathways.

### Metabolite extraction

Each sample consisted of 100 mg of tissue, which was ground with liquid nitrogen, followed by resuspension in prechilled 80% methanol and 0.1% formic acid by vortexing. The samples were incubated on ice for 5 min and then centrifuged at 15,000 rpm and 4°C for 5 min. A portion of the supernatant was diluted to a final concentration containing 53% methanol with LC-MS grade water. The samples were subsequently transferred to a fresh Eppendorf tube and centrifuged at 15,000 g and 4°C for 10 min. Finally, the supernatant was injected into an LC-MS/MS system for analysis. Liquid sample (100 μL) and prechilled methanol (400 μL) were mixed by vortexing, then mixed with prechilled 80% methanol by vortexing, followed by sonication for 6 min.

### UHPLC-MS/MS analysis

UHPLC-MS/MS analyses were performed using a Vanquish UHPLC system (Thermo Fisher, Germany) coupled with an Orbitrap Q ExactiveTM HF mass spectrometer (Thermo Fisher, Germany) at Novogene Co., Ltd. (Beijing, China). Samples were injected into a Hypersil Gold column (100×2.1 mm, 1.9 μm) using a 17-min linear gradient at a flow rate of 0.2 mL/min. The eluents for the positive polarity mode were eluent A (0.1% FA in water) and eluent B (methanol). The eluents for the negative polarity mode were eluent A (5 mM ammonium acetate, pH 9.0) and eluent B (methanol). The solvent gradient was set as follows: 2% B, 1.5 min; 2-100% B, 12.0 min; 100% B, 14.0 min; 100-2% B, 14.1 min; and 2% B, 17 min. A Q ExactiveTM HF mass spectrometer was operated in positive/negative polarity mode with a spray voltage of 3.2 kV, capillary temperature of 320°C, sheath gas flow rate of 40 arb, and aux gas flow rate of 10 arb.

### Data processing and metabolite identification

The raw data files generated by UHPLC-MS/MS were processed using Compound Discoverer v3.1 software (CD3.1, Thermo Fisher) to perform peak alignment, peak picking, and quantitation for each metabolite. The main parameters were set as follows: retention time tolerance, 0.2 min; actual mass tolerance, 5 ppm; signal intensity tolerance, 30%; signal/noise ratio, and minimum intensity, 100,000. Next, peak intensities were normalized to the total spectral intensity. The normalized data were used to predict the molecular formula based on additive ions, molecular ion peaks, and fragment ions. Peaks were then matched against the mzCloud (https://www.mzcloud.org/ ), mzVault, and MassList databases to obtain accurate qualitative and relative quantitative results. Statistical analyses were performed using the statistical software R v3.4.3, Python v2.7.6, and CentOS (release 6.6). When data were not normally distributed, normal transformations were attempted using the area normalization method.

### Data analysis

Metabolites were annotated using the KEGG database (https://www.genome.jp/kegg/pathway.html ) (Kanehisa and Goto, 2000), the human metabolome database (HMDB, https://hmdb.ca/metabolites ), and the LIPID Maps database (http://www.lipidmaps.org/ ). Principal component analysis (PCA) and partial least squares discriminant analysis (PLS-DA) were performed with MetaX. We applied univariate analysis (*t*-test) to calculate the statistical significance (*P*-value).

## Results

### Global transcriptomic differences in resistant and sensitive highland barley after pyroxsulam treatment

**Figure 1:**
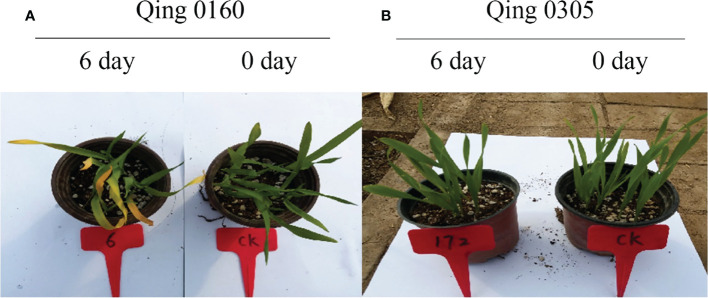
The different responses of the two barley varieties after pyroxsulam treatment. **(A)** The responses of the Qing 0160 after pyroxsulam treatment. **(B)** The responses of the Qing 0305 after pyroxsulam treatment.

**Figure 2:**
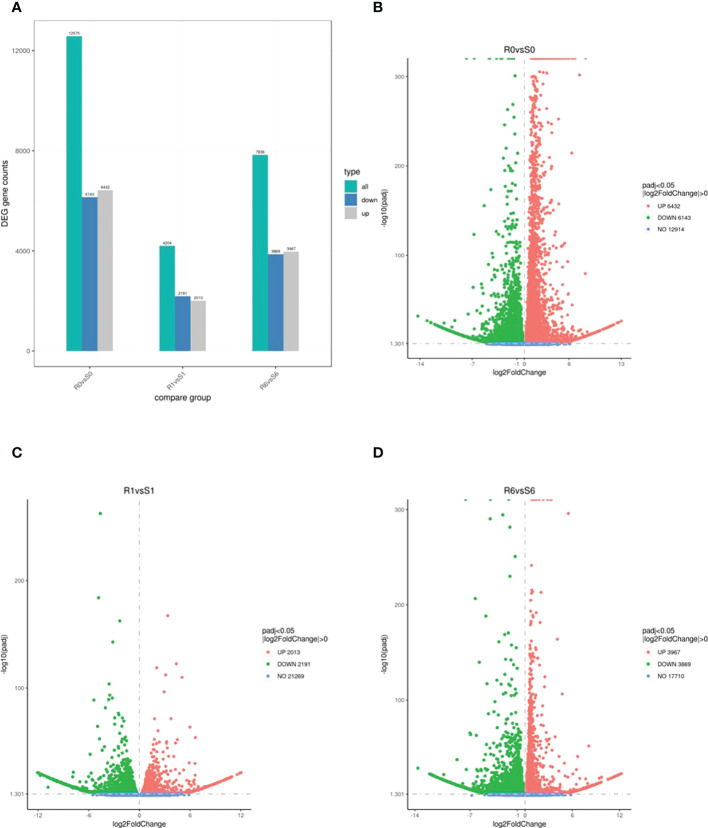
The number of DEGs down- and up-regulated in the resistant and sensitive highland barley genotypes after pyroxsulam treatment. **(A)** DEG counts of highland barley transcriptomes. **(B-D)**. Volcano maps of DEGs in the R0*vs*S0, R1*vs*S1 and R6*vs*S6 groups, respectively.

The two highland barley genotypes were exposed to pyroxsulam, and responses were measured at zero and six days after treatment. This analysis indicated that Qing 0160 plants were more tolerant compared to Qing 0305 plants (**Figure 1**). In order to determine the different responses of the two barley varieties at the molecular level, we compared transcriptomic data of resistant and susceptible barley at zero, one, and six days after pyroxsulam treatment. The resistant barley samples were named R0, R1, and R6, while the sensitive barley samples were named S0, S1, and S6. Raw and clean reads were obtained from 18 samples using the Illumina sequencing platform. We applied the fragments per kilobase of transcript per million mapped reads (FPKM) approach to normalize the expression levels of the genes. The numbers of DEGs in highland barley with more than a 2-fold change in expression (*P* < 0.05) when comparing R0 versus (*vs*) S0, R1 *vs* S1, and R6 *vs* S6 were then calculated. The numbers of DEGs varied between treatments (**Figure 2A**), and comparison of the two genotypes without pyroxsulam treatment resulted in the largest number of DEGs (6432 up-regulated and 6143 down-regulated genes in R0 *vs* S0) (**Figure 2B**). The comparison after one day of pyroxsulam treatment had the smallest number of DEGs (2013 up-regulated and 2191 down-regulated genes in R1 *vs* S1) (**Figure 2C**). The number of DEGs after 6 days of pyroxsulam exposure was slightly higher than 0 days after treatment (3967 up-regulated and 3869 down-regulated genes in R6 *vs* S6) (**Figure 2D**). These results indicated that different lengths of pyroxsulam treatment resulted in different gene expression profiles.

### Gene ontology enrichment analysis of DEGs in two highland barley genotypes with differing tolerance to pyroxsulam

**Figure 3:**
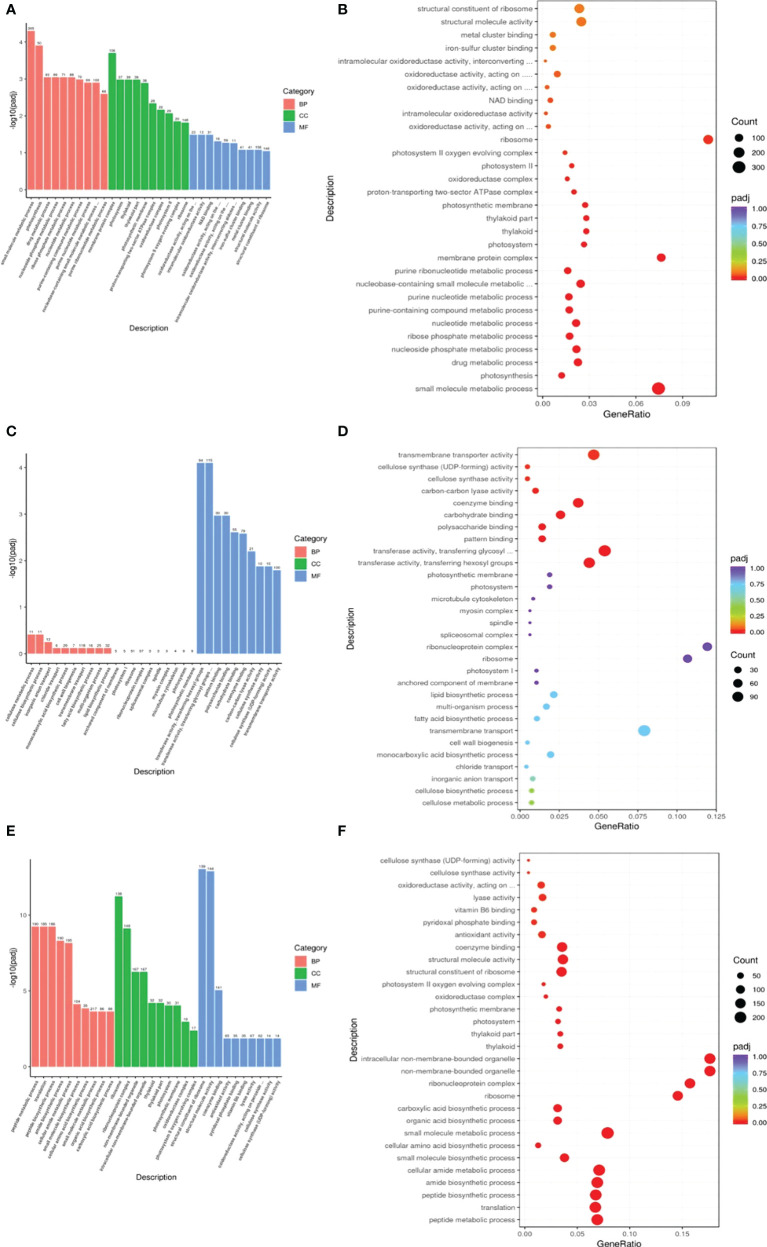
GO functional classification of the DEGs identified in this study. **(A, C, E)** Differential GO enrichment between the R0*vs*S0, R1*vs*S1 and R6*vs*S6 transcriptomes. **(B, D, F)** Significant GO enrichment in R0*vs*S0, R1*vs*S1 and R6*vs*S6, respectively.

DEGs were subjected to GO enrichment analysis to identify enriched biological functions. Genes associated with the small mol/ecule metabolic process, carbohydrate metabolic process and organic acid metabolic process showed the greatest differential expression in the R0*vs*S0 group in the biological process (BP) category, while genes associated with NAD binding and oxidoreductase activity, acting on the aldehyde or oxo group of donors showed the greatest differential expression in the R0*vs*S0 group in the molecular function (MF) category. Genes associated with transferase activity, transferring glycosyl groups, transmembrane transporter activity and transferase activity, and transferring hexosyl groups showed the greatest differential expression in the R1*vs*S1 group in the MF category. Genes associated with the small molecule metabolic process, cellular amide metabolic process and peptide metabolic process showed the greatest differential expression in the R6*vs*S6 group in the BP category. Genes associated with non-membrane-bounded organelle and intracellular non-membrane-bounded organelle and ribonucleoprotein complex showed the greatest differential expression in the R6*vs*S6 group in the cellular component (CC) category. Finally, genes associated with structural molecule activity, coenzyme binding and structural constituent of ribosome showed the greatest differential expression in the R6*vs*S6 group in the MF category (**Figure 3**). These data indicate that there are significant differences in resistant and sensitive highland barley varieties in a variety of biological processes, molecular functions and cellular components in response to pyroxsulam, which likely drive their different tolerances to this herbicide.

### KEGG pathway classification of DEGs in two highland barley genotypes with differing tolerance to pyroxsulam

**Figure 4:**
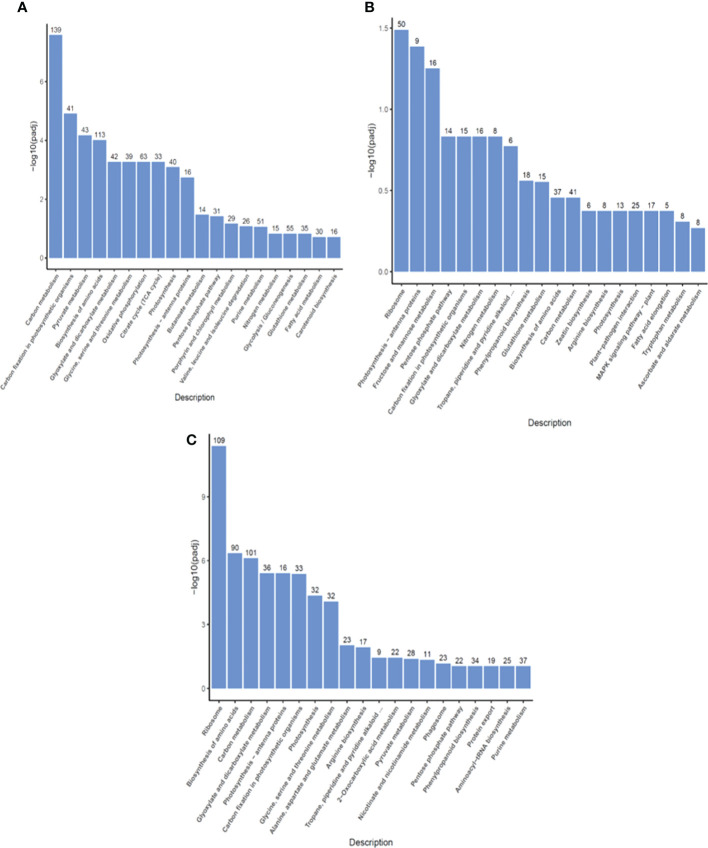
KEGG pathway classification of DEGs in highland barley. The most significant KEGG pathways in the R0 *vs* S0 group **(A)**, R1 *vs* S1 group **(B)**, and R6 *vs* S6 group **(C)**.

To identify what biological pathways the DEGs were associated with, KEGG pathway analysis was performed. In the primary metabolism category, DEGs from the R0 *vs* S0 comparison were enriched in pyruvate metabolism, biosynthesis of amino acids, and citrate cycle (**Figure 4A**). This enrichment indicated that DEGs of resistant and sensitive highland barley were associated with a wide range of basic biological processes involved in the maintenance of cell functions. DEGs associated with phenylpropanoid biosynthesis, glutathione metabolism, and tropane, piperidine and pyridine alkaloid biosynthesis were enriched in R1 *vs* S1 (**Figure 4B**). After one day of pyroxsulam treatment, DEGs were enriched for antioxidant-related genes in the glutathione metabolism pathway, and genes associated with stress in the phenylpropanoid and pyridine alkaloid biosynthesis pathways. In the R6 *vs* S6 comparison, the DEGs were also significantly enriched in the phenylpropanoid biosynthesis and the tropane, piperidine and pyridine alkaloid biosynthesis pathways. Additionally, pathways associated with biosynthesis and metabolism of amino acids were also significantly enriched (**Figure 4C**). Taken together, these results indicate that resistant and susceptible barley varieties have significantly different amino acid metabolism and stress responsive-gene profiles both with and without pyroxsulam treatment. The results shown that the DEGs of resistant and sensitive highland barley were enriched as a major flow from the genes of basic biological processes to increased stress resistant (redox metabolism and secondary metabolism) pathway during the growth process from 0, 1 to 6 days. The result indicated that the idea that resistant varieties may be “primed” to respond to the stress exerted by pyroxsulam treatment.

### Identification of differential metabolites of resistant and sensitive highland barley after pyroxsulam treatment

**Figure 5:**
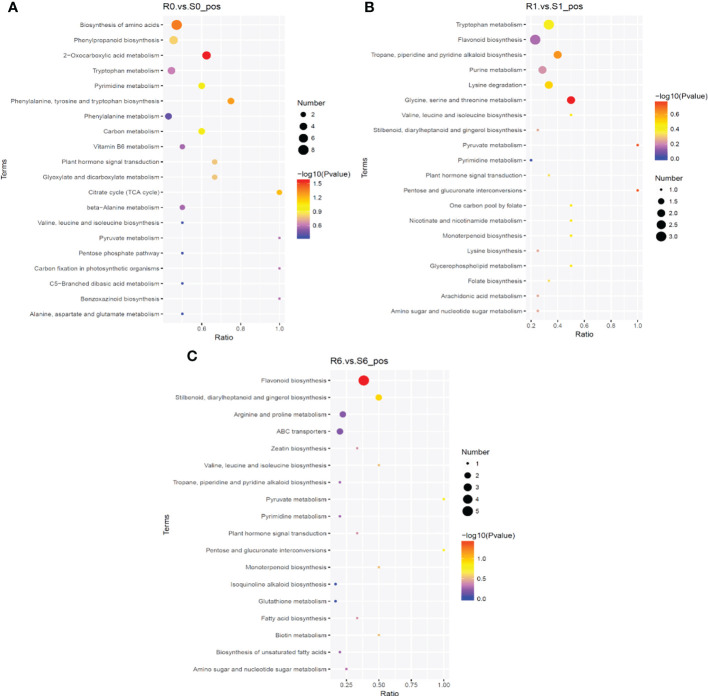
KEGG enrichment of differential metabolites. The abscissa is the ratio of the number of differential metabolites in the corresponding metabolic pathway to the total number of metabolites identified in the pathway. The larger the value, the higher the enrichment of differential metabolites in the pathway. The color of the dot represents the *P*-value of the hypergeometric test. The smaller the value, the greater the reliability of the test and the more statistically significant it is. The size of the dot represents the number of different metabolites in the corresponding pathway. The larger the dot, the more differential metabolites were found in the pathway. The most significant differential metabolites in the KEGG pathway in the R0 *vs* S0 group **(A)**, R1 *vs* S1 group **(B)**, and R6 *vs* S6 group **(C)**.

To compare the metabolic changes that took place after pyroxsulam treatment in resistant and sensitive highland barley after pyroxsulam treatment, metabolomic analysis of resistant and sensitive highland barley at zero, one, and six days of pyroxsulam treatment was conducted. In total, 837 and 386 metabolites in the positive and negative ionization modes were identified across all samples. To understand the metabolic activities of the two highland barleys after pyroxsulam treatment, we mapped the annotated metabolites to KEGG pathways. Enrichment analysis of the KEGG pathways indicated that biosynthesis of amino acids, citrate cycle, and phenylpropanoid biosynthesis were significantly enriched in the R0 *vs* S0 comparison (**Figure 5A**). Flavonoid biosynthesis and tropane, piperidine and pyridine alkaloid biosynthesis pathways were also significantly different in R1 *vs* S1 (**Figure 5B**). The glutathione metabolism pathway was also enriched in the R6 *vs* S6 comparison (**Figure 5C**). These results are in agreement with the transcriptomic data and further support the idea that amino acid metabolism and stress response genes underly the different pyroxsulam tolerances of the two barley varieties.

### Correlation between differential gene and differential metabolite expression

**Figure 6:**
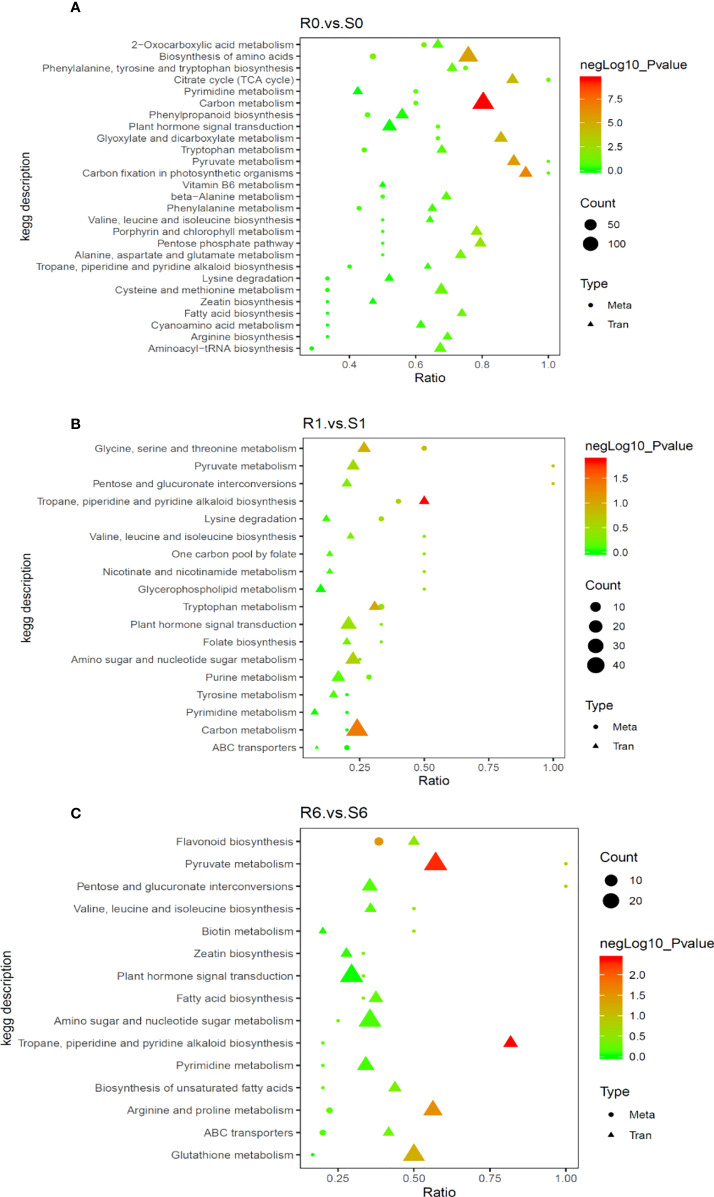
Correlation analysis of differentially expressed genes and differential metabolites in different KEGG pathways. **(A)** R0 vs S0 group, **(B)** R1 vs S1 group and **(C)** R6 vs S6 group. The abscissa is the ratio of the differential metabolites or differentially expressed genes enriched in the pathway to the number of metabolites or genes annotated in the pathway, and the ordinate is the common enrichment of metabolome-transcriptome. The count is the number of metabolites or genes enriched in the pathway.

To understand the regulatory networks of the two highland barley genotypes in response to pyroxsulam, a correlation analysis was carried out with DEGs obtained by transcriptome analysis and the significantly different metabolites obtained by metabolomics analysis to construct an integrated metabolic map (**Figure 6**). This map showed that the pathways involved in amino acid metabolism were enriched significantly without pyroxsulam treatment (R0 *vs* S0) (**Figure 6A**). The gene expression and metabolic flux shifted from amino acid metabolism to pyruvate metabolism and tropane, piperidine and pyridine alkaloid biosynthesis soon after pyroxsulam treatment (R1 *vs* S1) (**Figure 6B**). The genes involved in the phenylpropanoid and glutathione metabolic pathways were up-regulated, leading to the increased accumulation of flavonoids and antioxidants after six days of pyroxsulam treatment (R6 *vs* S6) (**Figure 6C**). Overall, these results indicate that several genes encoding key enzymes involved in the biosynthesis process of flavonoids and other crucial metabolites were differentially expressed, suggesting that these genes could be valuable targets for improved pyroxsulam tolerance in highland barley.

### Validation of gene expression using qRT-PCR

**Figure 7:**
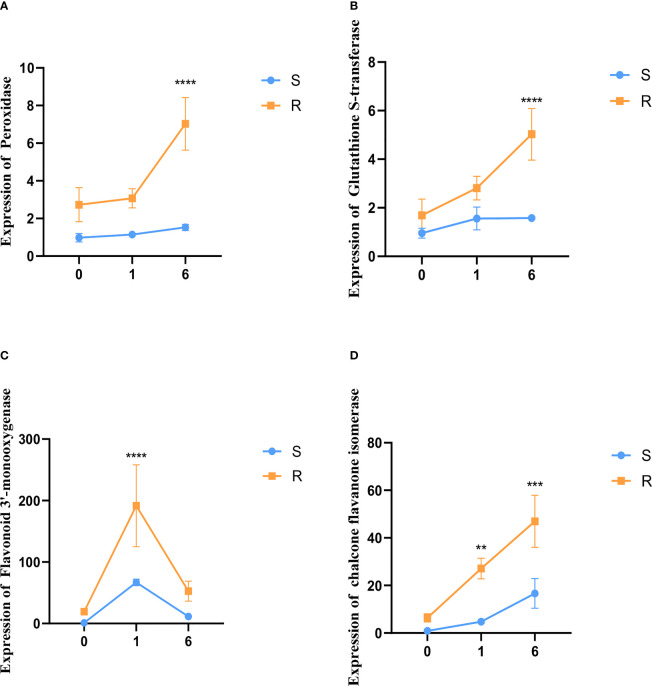
Validation of gene expression using qRT-PCR. **(A–D)** The expression levels of peroxidase, glutathione S-transferase, flavonoid 3’-monooxygenase, and chalcone flavanone isomerase were measured in the resistant barley and sensitive barley after 0, 1, and 6 days of pyroxsulam treatment (**P < 0.01, ***P < 0.001, and ****P < 0.0001).

Four genes which were differentially expressed in the transcriptome analysis were analyzed *via* qRT-PCR. This set was comprised of two genes involved in antioxidant pathways and two genes in flavonoid synthesis pathways. The qRT-PCR indicated that the expression levels of the antioxidant genes peroxidase (*PO*) and glutathione S-transferase (*GST*) were up-regulated in the resistant barley compared to sensitive barley after pyroxsulam treatment. Moreover, the level of expression increased further as the treatment time progressed in the pyroxsulam-resistant barley. However, the expression changes seen in *PO* and *GST* in the pyroxsulam-sensitive barley during treatment were not recapitulated in the qRT-PCR results (**Figures 7A**, **7B**). In addition, the expression level of the flavonoid 3’-monooxygenase was found to reach its maximum at one day after pyroxsulam treatment in both the resistant barley and sensitive barley. However, the expression level of the chalcone flavanone isomerase gene reached a maximum after six days of pyroxsulam treatment in both types of barley. In keeping with the transcriptome analysis, the expression levels of flavonoid 3’-monooxygenase and chalcone flavanone isomerase, which are key enzymes in the flavonoid synthesis pathways, were significantly higher in the resistant barely compared to the sensitive barley after pyroxsulam treatment (**Figures 7C**, **7D**). Overall, these results indicate that the higher tolerance of the pyroxsulam resistant barley variety could be linked to increased expression of antioxidant and flavonoid synthesis genes.

## Discussion

Weeds pose a significant threat to the production of highland barley, and new herbicides are required to deal with this ever-evolving problem (Powles and Yu, 2010). Advances in biotechnology have led to the discovery of several herbicides that belong to different chemical classes and possess diverse modes of action. These newer herbicides represent the most cost-effective and environmentally sustainable way to control weeds and increase worldwide food production (Green and Owen, 2011). Previously, herbicides could only be applied to a limited number of crops, but the development of herbicide-resistant varieties has greatly expanded their spectrum of use (Green, 2009; Gulden et al., 2009). Highland barley has not been extensively investigated for herbicide tolerance, which limits the herbicides that can be used to control weeds in barley fields. In this study, we examined two highland barley genotypes that differ significantly in pyroxsulam tolerance. We utilized time-course metabolic profiling and transcriptional profiling of tolerant and sensitive highland barley accessions to better understand the mechanisms underlying their tolerance differences. An integrated analysis of the transcriptome and metabolome revealed significantly more stress-responsive genes and metabolites in the pyroxsulam-resistant highland barley compared to pyroxsulam-susceptible barley. The relative expression of antioxidant-related genes involved in glutathione metabolism was significantly increased in the pyroxsulam-resistant highland barley compared to pyroxsulam-susceptible barley and led to accumulation of antioxidants. Several unique metabolites and unidentified compounds, such as glutathione, oxyresveratrol, and artemtherin, could play a protective role during responses to pyroxsulam. Taken together, these results indicate that highland barley responds to pyroxsulam treatment by inducing changes in antioxidants.

Flavonol metabolism has been reported to play a critical role in plant responses to a wide range of stressful conditions (Fini et al., 2011). Additionally, metabolic reprogramming of the phenylpropanoid pathway has been reported to play a key role in response to biotic and abiotic stresses in rice, maize, and soybean (Djamei et al., 2011; Tanaka et al., 2014; Dong et al., 2020). Metabolic profiling during fungal infection has revealed a mobilization of carbohydrates, changes in amino acid pools, and the activation of flavonoid biosynthetic pathways in soybean (Aliferis et al., 2014). Additionally, drought-stressed Arabidopsis has been reported to possess significantly different metabolite profiles, including large changes in flavonoids (Bechtold et al., 2016). It has previously been shown that flavonoids play a major role in protection against stresses in plants. For example, flavonoids accumulate in maize plants grown at high altitudes to prevent damage caused by high UV-B exposure (Casati and Walbot, 2005). Additionally, an increase in flavonoid secondary metabolites has also been shown to provide protection during pathogen infection (Ferreyra et al., 2012). Researchers have also reported that flavonoids are involved in the resistance to aluminum toxicity in maize (Kidd et al., 2001). Flavonoids have been shown to possess antimicrobial activity in plants and have potent antifungal activity against several major plant fungal pathogens, including *Fusarium oxysporum* (Gill et al., 2018). In the current study, we found that the expression of key genes in the phenylpropanoid pathway, and the biosynthesis of flavonoids significantly increased during pyroxsulam treatment in the pyroxsulam-resistant highland barley. The flavonoid content gradually increased with prolonged pyroxsulam treatment time, but the flavonoid content in the pyroxsulam-sensitive highland barley did not significantly change after pyroxsulam treatment. The pyroxsulam-tolerant barley had stronger induction of flavonoids, which may represent a mechanism responsible for its higher tolerance. These results provide a starting point for using metabolomics to assist in the breeding of pyroxsulam-tolerant highland barley varieties.

**Figure 8:**
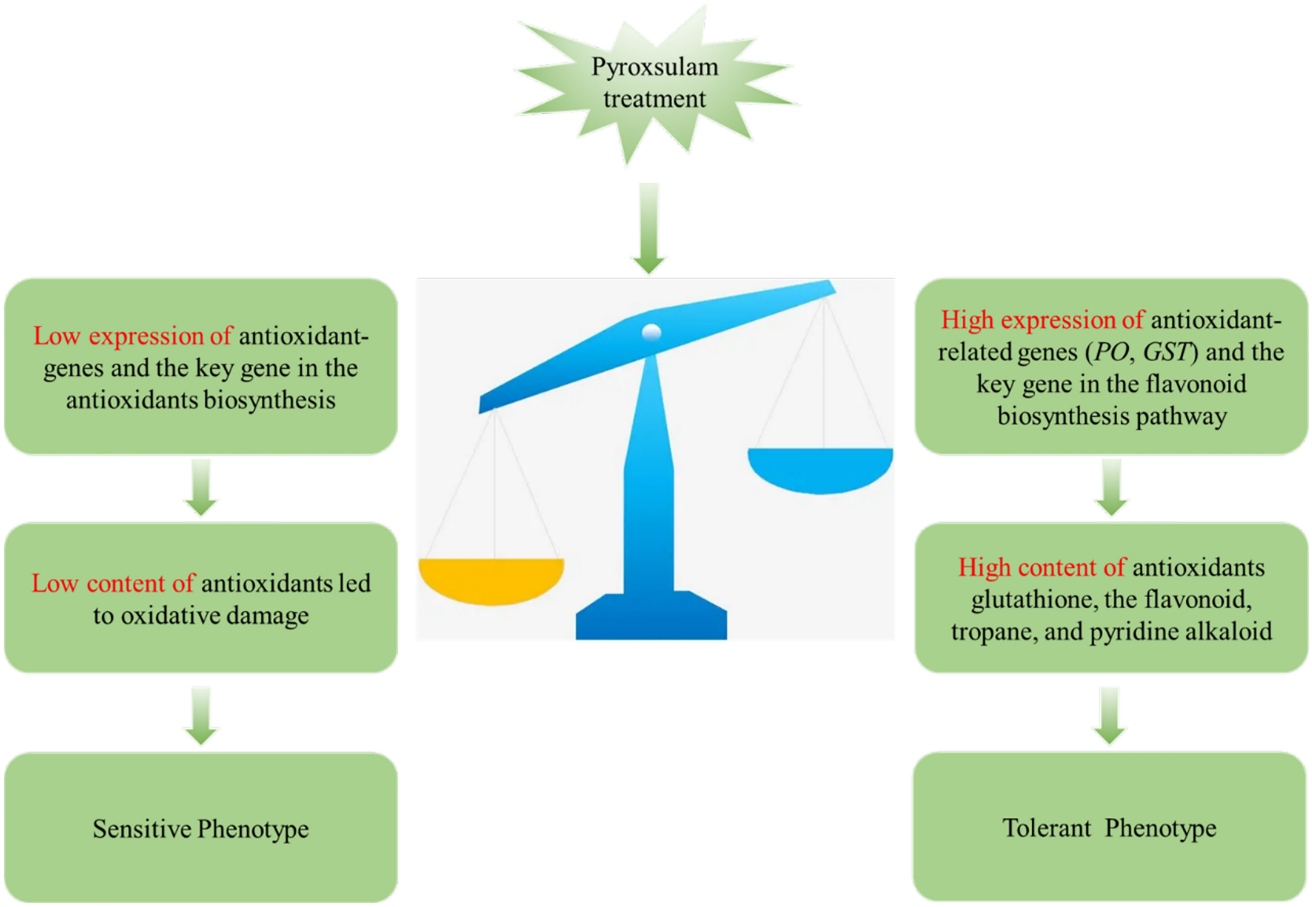
Schematic describing a hypothetical model of the mechanism by which two highland barley genotypes differing sensitivities to pyroxsulam.

Herbicides have been demonstrated to result in the generation of ROS in plants, with antioxidant systems often being responsible for the tolerance level of a given species (Caverzan et al., 2019). Thioredoxin reductase plays a key role in catalyzing the NADPH-dependent reduction of oxidized thioredoxin to scavenge ROS, and this gene has been shown to be induced during salt stress in hulless barley (Lai et al., 2020). Gene expression analysis in wheat revealed that most unigenes encoding peroxidase were induced by biotic stress, leading to better resistance to *Bipolaris sorokiniana* (Ye et al., 2019). In the current study, transcriptome analysis revealed that the gene expression levels of glutathione S-transferase and glutathione synthetase, both of which scavenge ROS, were significantly increased in the pyroxsulam-resistant highland barley genotype. In addition, metabolite analysis revealed that the metabolite glutathione is uniquely induced in the tolerant highland barley after treatment with pyroxsulam. These results demonstrate that genes associated with the antioxidant system were up-regulated, which led to accumulation of antioxidants and subsequent scavenging of ROS in the tolerant highland barley variety. In addition, the expression of key genes in the phenylpropanoid pathway and the biosynthesis of flavonoids significantly increased during pyroxsulam treatment in the pyroxsulam-resistant highland barley. Flavonoids are a class of specialized metabolites, with subclasses including flavonols and anthocyanins, which have unique properties as antioxidants that can scavenge ROS (Agati et al., 2012). It can be inferred from this observation that pyroxsulam exposure induces accumulation of flavonoids and antioxidant enzyme genes, resulting in increased ROS scavenging and enhanced pyroxsulam tolerance in highland barley. These pathways are not induced in the susceptible highland barely, which likely contributes to its pyroxsulam sensitivity (**Figure 8**). Our results shed new light on the molecular mechanisms underlying variable pyroxsulam tolerance in highland barley and are an important first step in the development of tolerant crops through gene editing or traditional breeding.

## Data availability statement

The original contributions presented in the study are publicly available. This data can be found here: [https://www.ebi.ac.uk/metabolights/index; accession number MTBLS3936].

## Ethics statement

Qing 0160 and Qing 0305 were obtained from Xining City, Qinghai Province, China (36.434419 N, 101.450759 E). The samples were identified by Liling Jiang and deposited at the National duplicate Genbank for crops, Xining, Qinghai Province, China. The specimen accession numbers are ZDM1651 and ZDM8091. The authors declare that they comply with the IUCN policy statement on Research Involving Species at Risk of Extinction and the Convention on the Trade in Endangered Species of Wild Fauna and Flora.

## Author contributions

HW and JY conceived the study and designed the experiments. HW and LG carried out the experiments. HW wrote the manuscript and created the charts and figures. HC guided and revised the article. All authors contributed to the article and approved the submitted version.
